# Intersociety Position Statement on the Prevention of Ophthalmia Neonatorum in Italy

**DOI:** 10.3390/microorganisms12010015

**Published:** 2023-12-21

**Authors:** Chryssoula Tzialla, Cinzia Auriti, Salvatore Aversa, Daniele Merazzi, Stefano Martinelli, Gabriella Araimo, Luca Massenzi, Giacomo Cavallaro, Luigi Gagliardi, Mario Giuffrè, Fabio Mosca, Irene Cetin, Vito Trojano, Herbert Valensise, Nicola Colacurci, Luigi Orfeo, Vito Mondì, on behalf of their respective Scientific Societies

**Affiliations:** 1 Neonatal and Pediatric Unit, Polo Ospedaliero Oltrepò, ASST Pavia, Via Volturno 14, 27058 Voghera, Italy; chryssoula.tzialla@unipv.it; 2 Faculty of Medicine, UniCamillus-Saint Camillus International University of Health Sciences, Via di Sant’Alessandro 8, 00131 Rome, Italy; 3 Neonatologic Unit, Villa Margherita Private Clinic, Viale di Villa Massimo 48, 00161 Rome, Italy; 4 Neonatal Intensive Care Unit, Children’s Hospital, ASST Spedali Civili, Piazzale Spedali Civili 1, 25123 Brescia, Italy; salvatore.aversa@asst-spedalicivili.it; 5 Division of Neonatology, ‘Valduce’ Hospital, Via Dante Alighieri 11, 22100 Como, Italy; dmerazzi@valduce.it; 6 Neonatal Intensive Care Unit, ASST Grande Ospedale Metropolitano Niguarda, Piazza dell’Ospedale Maggiore 3, 20162 Milan, Italy; stefanoenrico.martinelli@ospedaleniguarda.it; 7 Neonatal Intensive Care Unit, Fondazione IRCCS Ca’ Granda Ospedale Maggiore Policlinico, Via Francesco Sforza 28, 20122 Milan, Italy; gabriella.araimo@gmail.com (G.A.); giacomo.cavallaro@policlinico.mi.it (G.C.); fabio.mosca@unimi.it (F.M.); 8 Division of Neonatology, Central Teaching Hospital of Bolzano, Via Lorenz Böhler 5, 39100 Bolzano, Italy; lumass2001@yahoo.it; 9 Department of Mother and Child Health, Azienda USL Toscana Nord Ovest, Via A. Cocchi, 7/9, 56121 Pisa, Italy; luigi.gagliardi@uslnordovest.toscana.it; 10 Neonatal Intensive Care Unit, A.U.O.Policlinico ‘Paolo Giaccone’, Department of Health Promotion, Mother and Child Care, Internal Medicine and Medical Specialties ‘G. D’Alessandro’, University of Palermo, Via del Vespro 129, 90126 Palermo, Italy; mario.giuffre@unipa.it; 11 Department of Clinical Sciences and Community Health, University of Milan, Via della Commenda 19, 20122 Milan, Italy; 12 Department of BioMedical and Clinical Sciences, University of Milan, Via Gian Battista Grassi 74, 20122 Milan, Italy; irene.cetin@unimi.it; 13 Department of Obstetrics and Gynecology, Hospital V. Buzzi, ASST Fatebenefratelli Sacco, Via Lodovico Castelvetro 32, 20154 Milan, Italy; 14 Department of Obstetrics and Gynaecology, Mater Dei Hospital, Via Samuel F. Hahnemann 10, 70125 Bari, Italy; vtrojano@katamail.com; 15 Department of Surgical Sciences, University of Rome Tor Vergata, Via Montpellier, 1, 00133 Rome, Italy; valensise@med.uniroma2.it; 16 Obstetrics and Gynecology, Policlinico Casilino, Via Casilina 1049, 00169 Rome, Italy; 17 Department of Woman, Child and General and Specialized Surgery, University of Campania “Luigi Vanvitelli”, Via Luigi De Crecchio 2, 80138 Naples, Italy; nicola.colacurci@unicampania.it; 18 Neonatal Intensive Care Unit, Fatebenefratelli Isola Tiberina—Gemelli Isola, Via di Ponte Quattro Capi 39, 00186 Rome, Italy; orfeo@iol.it; 19 Neonatology and Neonatal Intensive Care Unit, Policlinico Casilino, Via Casilina 1049, 00169 Rome, Italy; vito.mondi@mail.com

**Keywords:** ophthalmia neonatorum, *Neisseria gonorrhoeae*, *Chlamydia trachomatis*, neonatal ocular prophylaxis

## Abstract

There is currently no worldwide agreement on the real need to administer conjunctival antibiotics to neonates at birth to prevent neonatal conjunctivitis (usually defined as ophthalmia neonatorum) by *Chlamydia trachomatis* and *Neisseria gonorrhoeae*. Therefore, there is wide variability in antibiotic administration, conditioned mainly by the social and health context. In Italy, a law enacted in 1940 required doctors and midwives to administer ophthalmic prophylaxis with 2% silver nitrate to all newborns at birth. This law was repealed in 1975 and since then there has been no clear guidance on the use of ophthalmia neonatorum prophylaxis at birth. Since neonatal conjunctivitis caused by *C. trachomatis* and *N. gonorrhoeae* is not reported, we carried out a nationwide survey of 1,041,384 neonates across all Italian birth centers to evaluate the incidence of ophthalmia neonatorum and the current practice of prophylaxis. After analyzing the results, we formulated an intersociety position statement on the prevention of ophthalmia neonatorum to update and standardize this prevention strategy in Italy.

## 1. Introduction

Ophthalmia neonatorum (ON) is an acute conjunctival infection that occurs within the first month of life [1]. The infection, usually contracted from the mother’s infected birth canal during vaginal delivery, can cause corneal damage and vision loss. In the past, the term mainly defined conjunctival infections caused by *Chlamydia trachomatis* and *Neisseria gonorrhea*, which are the pathogens most frequently responsible for permanent ocular damage [1,2]; currently, ON also defines neonatal conjunctivitis caused by other bacteria [3]. The incidence of ON by *N. gonorrhoeae* has significantly decreased with the widespread use of conjunctival antibacterial prophylaxis with 2% silver nitrate, introduced by Karl Franz Credè in Germany at the end of the 19th century [2,4,5]. While the updated incidence of ON by *C. trachomatis* and *N. gonorrhoeae* reported in the literature is 1.6–23%, the real incidence is unknown [6,7].

The main pathogen for ON varies between countries and depends on the socio-medical context of the observed population. In the United States, *C. trachomatis* is responsible for 2–40% of reported cases; *N. gonorrhoeae* is rare, accounting for less than 1% of cases, while other bacteria species account for 30–50% of cases [1,8]. *N. gonorrhoeae* is no longer the most common bacterial agent of ON across many countries, regardless of income [1,6]. Data from a survey conducted among 449 ophthalmologists from around the world, members of the American Association of Pediatric Ophthalmology and Strabismus, report that 176 of 291 responders (60.69%) experienced 0–5 cases of ON per year, with *C. trachomatis* accounting for up to 35.4% of cases and *N. gonorrhoeae* for 10% of cases [6]. Other bacteria, such as *Staphylococcus*, *Streptococcus* and *Haemophilus*, were responsible for almost 50% of cases, while 7% of infections were due to viral agents such as adenovirus and herpes simplex virus [6].

Recent data from Ireland show that over a 5-year period, the most frequent etiologic agent of ON was *C. trachomatis*, accounting for 20.4% of all reported cases, while no cases of gonococcal infection were reported [9]. In case series from Hong Kong, Iran, Norway and Ghana, the most frequent isolates were *Staphylococcus aureus* [10,11,12] or *Staphylococcus* spp. [7], while *C. trachomatis* accounted for 21% in Hong Kong, 2% in Iran and 0% in Norway and Ghana, and *N. gonorrhoeae* was uncommon (1–3%) or absent [7,8,11,12]. In Argentina, 56 out of 332 identified cases (16.8%) were due to *H. influenzae* and only 25 (7.8%) to *C. trachomatis* [13].

The rate of gonococcal or chlamydial conjunctivitis in neonates is related to the maternal infection rate. Gonococcal or chlamydial infection in women, even during pregnancy, is often asymptomatic and, as such, remains undiagnosed [14]. Transmission rates of gonococcal infection from mother to newborn, in the absence of maternal treatment and neonatal ocular prophylaxis, are 30–50% [2,15,16]. It is estimated that almost 20% of untreated, or inappropriately treated, gonococcal conjunctivitis can result in corneal damage [16,17].

The risk of *C. trachomatis* transmission from an untreated infected mother to newborn is 50%, with a 25–50% risk of developing conjunctivitis; the risk of developing pneumonia requiring systemic therapy is 5–20% [1,2,15]. Untreated chlamydial conjunctivitis can be associated with corneal and conjunctival scarring, hemorrhagic conjunctivitis and, rarely when compared with gonococcal conjunctivitis and loss of vision [10,18,19]. Topical eye prophylaxis does not prevent transmission from mother to newborn and does not prevent neonatal conjunctivitis and pneumonia [1,15,20].

Since Italy lacks specific regulations on the use of ocular prophylaxis for the prevention of ON, as well as data on the incidence of conjunctivitis caused by *C. trachomatis* and *N. gonorrhoeae*, we carried out a nationwide survey to evaluate the incidence of ON and the current use of prophylaxis. After the analysis of survey results, an intersociety position statement on the prevention of ON was formulated to update and standardize the use of ocular prophylaxis for ON in Italy.

## 2. Strategies for the Prevention of Ophthalmia Neonatorum and Current Guidelines

The interruption of maternal–neonatal infection transmission is the mainstay approach for the prevention of ON [1,2]; this can be achieved by:

1. Screening pregnant women for sexually transmitted infections (STIs) and giving appropriate treatment as necessary; treated patients and those with persistent risk factors for STI should be regularly followed up with and re-tested.
2. Ocular prophylaxis, with antiseptic or antibiotic medication, of the neonate after birth.

The Centers for Disease Control and Prevention (CDC) recommends routine screening for *C. trachomatis* and *N. gonorrhoeae* at the first prenatal visit for all high-risk women. The latter are defined as those being younger than 25 years of age, or older women at increased risk of infection (e.g., new or multiple sex partners, or a sex partner with concurrent partners or with an STI [21,22]) or living in an area in which the prevalence of *C. trachomatis* or *N. gonorrhoeae* is high. Women who remain at increased risk of STI should be rescreened during the third trimester of pregnancy; pregnant women identified as positive for *C. trachomatis* or *N. gonorrhoeae* should be treated immediately and re-tested 3 months after treatment, as well as in the third trimester [14,23].

The guidelines of the Italian Ministry of Health [24] recommend that screening for gonococcal and chlamydial infection should be offered to pregnant women at risk for STI (i.e., those under 25 years of age and sexually active, or with multiple sexual partners, or with previous episodes of STI and from areas with high disease prevalence) during the first prenatal assessment and third trimester.

The nucleic acid amplification test (NAAT) is the most sensitive and specific diagnostic test for the identification of *C. trachomatis* approved by the CDC, United States Preventive Services Task Force (USPSTF) and Food and Drug Administration (FDA) [22]. The CDC and USPSTF also support the use of NAAT for the detection of gonococcal infections, while the FDA approves its use for the diagnosis of genitourinary gonococcal infections; *N. gonorrhoeae* can also be detected by culture or Gram stain of male urethral swabs but, due to the lower sensitivity of Gram staining compared with NAAT, a negative result cannot rule out a gonococcal infection in an asymptomatic male [22].

The World Health Organization (WHO) guidelines [25] recommend ophthalmic prophylaxis for all newborns at birth by the administration of one of the following drugs into both eyes:

- Tetracycline hydrochloride 1% eye ointment;
- Erythromycin 0.5% eye ointment;
- Povidone iodine 2.5% solution (water based);
- Silver nitrate 1% solution;
- Chloramphenicol 1% eye ointment.

The choice of drug depends on the local availability of the molecule and cost.

According to the WHO, the benefits of the above intervention outweigh the potential risk of chemical conjunctivitis; silver nitrate effectively prevents gonococcal ON, but its use has been restricted because it causes transient chemical conjunctivitis [2,26].

Currently, there is no universal agreement on the need for conjunctival prophylaxis at birth, and practice varies widely around the world in relation to the social and health context. Taking into account WHO recommendations, each nation has issued specific guidelines based on its own profile. Countries that perform ON prophylaxis to all neonates at birth include Brazil [27], USA [16,28], Spain [29], Slovenia [30], Turkey [31], Ghana [7] and Chile [32]; prophylaxis is also undertaken in certain areas of Central America, some countries in Africa, parts of the Far East, areas of the Middle East and parts of Central Asia [6]. Conversely, other countries with well-organized systems of prenatal care, such as Denmark, the Netherlands, Norway, Sweden, France, Belgium, the UK, Canada and Australia, recommended the discontinuation of ocular prophylaxis several years ago [4,13,15]. Despite the withdrawal of the practice, the resurgence of gonococcal ON or associated blindness has not been reported [33,34].

In Italy, despite the 1940 law rendering prophylaxis compulsory [35] being repealed in 1975 [36], the administration of conjunctival antibiotics to newborns is still routinely carried out.

## 3. Incidence of Gonorrhea and *C. trachomatis* Infections in Italy

Data from the Italian Institute of Health (Istituto Superiore di Sanità [ISS]) report 10,597 new cases of gonorrhea in Italy between 1991 and 2021, with a three-fold increase between 2010 and 2021, mostly between homosexual males [37]. A higher frequency of gonococcal infection was observed in males (94.1% vs. 5.9% among females), individuals aged between 15 and 24 years, foreign nationals and those with more than one sexual partner [37]. In the latest report from the European Centre for Disease Prevention and Control, 117,881 cases of gonorrhea with an incidence of 31.6 cases per 100,000 population were reported in Europe in 2019; 813 cases were reported in Italy, equal to an incidence of 1.3 cases per 100,000 inhabitants, among the lowest in Europe [38].

Regarding *C. trachomatis* infections, data from the ISS report 11,383 new cases from 1 January 1991 to 31 December 2021 [37]; the majority of cases were diagnosed in men (69.4% vs. 30.6% in women). Approximately one fifth of cases (20.3%) were foreign nationals, mainly from other European countries and Africa (48.7% and 28.5%, respectively); over half of subjects (55.6%) reported having had two to five sexual partners in the previous six months, and 15.5% had six or more. Regarding the mode of transmission, 51.4% of cases were reported in heterosexual males, 16.4% in homosexual males and 32.2% in females [37]. Unfortunately, cases of neonatal conjunctivitis due to *N. gonorrhoeae* and *C. trachomatis* are not subjected to specific surveillance and notification in Italy and therefore remain unreported [39].

Therefore, given that there is no legislation in force or national guidelines from Italian scientific societies [40], we conducted a nationwide questionnaire survey to collect data regarding the incidence of gonococcal and chlamydial conjunctivitis in neonates in addition to the type of medication used for ON prophylaxis (i.e., type of molecule, the concentration of active ingredient, composition and packaging of the drug) compared with WHO recommendations.

The questionnaire, in electronic format, was sent to all Italian birth centers and the data were collected over a three-year period (2018–2020). Three hundred and two of the 419 centers (72%) replied to the survey, which included details of 1,041,384 neonates, corresponding to 82.3% of those born in Italy during the period of the study (Table 1). In those three years, all newborns born in the participating centers underwent ocular prophylaxis with antibiotics in the first hours of life. Among them, 99.6% did not receive prophylaxis according to the WHO recommendations, both in terms of type of molecule, concentration of active ingredient, preparation and composition (multiple or single active ingredients) (Table 2). Chloramphenicol and tetracycline were administered to 3.6% and 4.4% of newborns, respectively, but with a different preparation (eye drops) and composition (a combination of more than one active drug) compared with WHO recommendations (Table 2). A single-dose package, strongly recommended to prevent cross-infections, was used only in 54% of newborns. Concerning the incidence and etiology of conjunctivitis during the period of the study, 12 cases of conjunctivitis caused by *C. trachomatis* were observed, giving an incidence of 0.001%, while no cases of gonococcal conjunctivitis were reported.

A detailed description of the survey methodology, including questionnaire composition, identification of participants and data collection and analysis, is part of a previous publication [40].

## 4. Intersociety Recommendations on Ophthalmia Neonatorum Prophylaxis in Italy

Considering the results of the survey, which highlighted a low incidence of neonatal conjunctivitis from *C. trachomatis* and *N. gonorrhoeae* in Italy, and, given the lack of local legislation on ocular prophylaxis for all neonates at birth, the Italian Society of Neonatology, of Gynecology and Obstetrician and Perinatal Medicine formulated an intersociety position statement.

The goal of that document is to provide guidance towards an optimized prevention strategy for ON that avoids unnecessary antibiotic administration.

The boards of the respective societies designated expert members from each specialist area to develop the policy statement. The expert panel undertook a literature search and identified current published recommendations, using the findings to inform the first version of the intersociety statement. The initial draft statement was then circulated among the board members of the three scientific societies to elicit individual comments for improvement. A final Italian-language version of the policy statement, addressing all comments and inclusive of proposed modifications, was endorsed by the respective board members. The final document was published on the scientific society websites.

**Figure 1:**
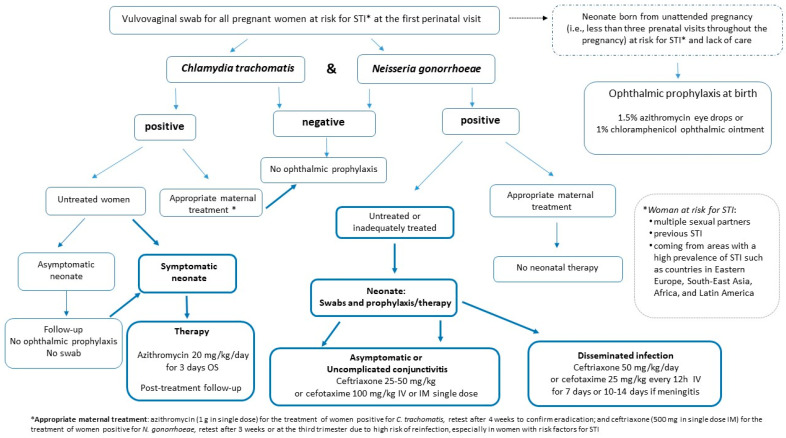
Recommendations flow chart [22,23,25,28,42,43,44,45,46,47].

Recommendations (Figure 1):

1. Do not give conjunctival antibiotic prophylaxis against ON indiscriminately to all infants at birth.
2. Ophthalmic prophylaxis with 1.5% azithromycin eye drops or 1% chloramphenicol ophthalmic ointment [25,28,42] should be administered immediately after birth exclusively to neonates born from unattended pregnancies (defined as fewer than three prenatal visits throughout the pregnancy); mothers at risk of sexually transmitted diseases, or coming from areas with a high prevalence of gonococcal infections and with no access to care; and when it is assumed that no primary prevention of STI has taken place before, and during, pregnancy. Maternal screening for *N. gonorrhoeae* and *C. trachomatis* at delivery, or immediately postpartum, should be performed.
3. Screening of women at risk of STI should be performed at the first prenatal care visit by a vulvovaginal swab for *N. gonorrhoeae* and *C. trachomatis* [22,23]. Swabs should be repeated in the third trimester in the case of ongoing exposure to risk (i.e., women with multiple sexual partners, previous STI, or coming from areas with a high prevalence of the disease such as Eastern Europe, South East Asia, Africa and Latin American countries). Women without risk factors who have not been swabbed during pregnancy and who have been regularly followed-up with by the obstetrician are to be considered negative.
4. Screening for *N. gonorrhoeae* and *C. trachomatis* at delivery, or immediately postpartum, should be performed in women with risk factors for STI who, despite having access to care and being followed-up with during pregnancy, have not been screened, to ensure appropriate management of the mother–neonate in the event of a positive test [28]. Pending the result of the maternal swab, the asymptomatic newborn can be routinely discharged from the birthplace without receiving conjunctival antibiotics. The mother should be recalled to communicate the result of the vaginal swab. Neonates, even if asymptomatic, born to mothers with a vulvovaginal swab positive for *N. gonorrheae*, or those with conjunctival and/or respiratory symptoms born to mothers with a swab positive for *C. trachomatis*, must be re-evaluated by the pediatrician to ensure appropriate therapy (see recommendation numbers 7–9).
5. Neonates born to women at risk for STI who were not screened during pregnancy, or to those positive for *C. trachomatis*, even untreated, in the absence of clinical symptoms of conjunctivitis, should not undergo an ocular swab.
6. In the absence of conjunctival and/or respiratory symptoms, neonates born to women infected by *C. trachomatis* should not undergo ophthalmic prophylaxis or systemic therapy; instead, they should be subject to pediatrician follow-up to recognize early on any signs of conjunctivitis, which usually occurs between 5 and 14 days of life, or pneumonia, which has a later onset of between 4 and 12 weeks [28].
7. Symptomatic neonates with conjunctival and/or respiratory signs born to mothers with untreated chlamydial infection should be treated with oral azithromycin at a dose of 20 mg/kg/day, once a day, for 3 days [42,43,44,45]. Follow-up is recommended to determine the effectiveness of the initial treatment because data on the efficacy of azithromycin for eye or lung disease are limited [43,44].
8. Asymptomatic neonates born to women with untreated or inadequately treated *N. gonorrhoeae* infection [45] should be evaluated by swab (e.g., ocular, rectal, vaginal and oropharyngeal). Treatment should start immediately with 25–50 mg/kg of ceftriaxone intramuscularly (IM) or intravenously (IV), up to a maximum of 250 mg total, or cefotaxime 100 mg/kg IM or IV in a single dose when ceftriaxone is contraindicated (i.e., concomitant treatment with calcium-containing IV fluids), pending the results of the swabs [23,46,47].
9. Symptomatic neonates with:
  - Uncomplicated gonococcal conjunctivitis should be evaluated to rule out disseminated gonococcal infection and treated with a single dose of ceftriaxone, 25–50 mg/kg IM or IV, or a single dose of cefotaxime 100 mg/kg, IM or IV, when ceftriaxone is contraindicated (i.e., concomitant treatment with calcium-containing IV fluids) [23,46,47].
  - Disseminated gonococcal infection should be hospitalized and receive IV therapy with ceftriaxone 50 mg/kg every 24 h (or cefotaxime 25 mg/kg every 12 h) for 7 days, or for 10–14 days in the case of documented meningitis [23,46,47].

## 5. Conclusions

Given the low incidence of conjunctival infections from *C. trachomatis* and *N. gonorrhoeae* in the newborns observed in Italy throughout the survey conducted between 2018 and 2020, and in the absence of current legislation determining the prophylaxis of ON, the Italian societies SIN, SIGO and SIMP have drawn up a position statement that eliminates the obligatory administration of conjunctival antibiotics in Italy to all neonates at birth. Instead, the position statement recommends starting therapy in newborns in which conjunctival and/or respiratory, and/or systemic symptoms, suggestive of *C. trachomatis* or *N. gonorrhoeae* infection occur. Simultaneously, it is necessary to implement prenatal screening for STIs in pregnant women at their first prenatal visit. This approach enables the treatment of infected mothers while avoiding the inappropriate administration of antibiotics to neonates at birth.

## Figures and Tables

**Table 1:** Geographical distribution of total respondent Italian birth centers, subdivided by the number of assisted births in 2020 [41].

	Northern Italy	Central Italy	Southern Italy and Islands	Total
Birth centers	173	89	157	419
Respondent centers (%)	137 (79.2)	63 (70.8)	102 (65)	302 (72.3)
N° of birth centers with <500 births/year	38	24	41	103
N° of respondent centers with <500 births/year (%)	19 (50)	13 (54.2)	18 (43.9)	50 (48.5)
N° of birth centers with 500–999 births/year	67	37	66	170
N° of respondent centers with 500–999 births/year (%)	56 (83.6)	26 (70.3)	41 (62.1)	123 (72.3)
N° of birth centers with 1000–2499 births/year	55	23	49	127
N° of respondent centers with 1000–2499 births/year (%)	51 (92.7)	19 (82.6)	42 (85.7)	112 (88.2)
N° of birth centers with >2500 births/year	13	5	1	19
N° of respondent centers with >2500 births/year (%)	11 (84.6)	5 (100)	1 (100)	17 (89.5)

**Table 2:** Comparison between WHO recommendations and ON prophylaxis medications used in the three years (2018–2020) of observation in Italy: type of active ingredient, multiple or single active ingredient, preparation, packaging.

	% of Neonates	Preparation	Composition	WHO Recommendations
**Active ingredient**				
Tobramycin 0.3%	45.6%	Eye drops	Single active drug	Not recommended
Gentamicin 0.3%	19.5%	Eye drops	Single active drug	Not recommended
Netylmycine 0.3%	11.3%	Eye drops	Single active drug	Not recommended
Ofloxacin 0.3%	11%	Eye drops	Single active drug	Not recommended
Chloramphenicol	3.6%	Eye drops	Combination of active drugs	Recommended ointment as single active drug
Tetracycline 1%	4.4%	Ointment	Combination of active drugs	Recommended as single active drug
Fusidic acid 1%	3.1%	Eye drops	Single active drug	Not recommended
Povidone iodine 2.5%	0.4%	Water solution	Single active drug	Recommended
Auromycin 1%	0.1%	Ointment	Single active drug	Not recommended
Pheniramine maleate 0.3% + tetrazoline hydrochloride 0.05%	0.6%	Eye drops	Combination of active drugs	Not recommended
Azithromycin 1.5%	0.4%	Eye drops	Single active drug	Not recommended
**Packaging**				
Single-use package	54% of treated neonates			
Multi-use package	46% of treated neonates			

## Data Availability

The data that support the findings of this study are available from the corresponding author, Cinzia Auriti upon reasonable request.
